# Nation-wide epidemiological study of Japanese patients with rare viral myelopathy using novel registration system (HAM-net)

**DOI:** 10.1186/s13023-016-0451-x

**Published:** 2016-05-25

**Authors:** Ariella L. G. Coler-Reilly, Naoko Yagishita, Hiroko Suzuki, Tomoo Sato, Natsumi Araya, Eisuke Inoue, Ayako Takata, Yoshihisa Yamano

**Affiliations:** Department of Rare Diseases Research, Institute of Medical Science, St. Marianna University School of Medicine, Kawasaki, Kanagawa Japan; Center for Clinical Research and Development, National Center for Child Health and Development, Tokyo, Japan; Department of Preventive Medicine, St. Marianna University School of Medicine, Kawasaki, Kanagawa Japan

**Keywords:** HTLV-1, HAM/TSP, OMDS, HAQ-DI, Registry, Japan, Blood transfusion, Family history

## Abstract

**Background:**

At least one million people are infected with human T-lymphotropic virus type 1 (HTLV-1) in Japan, a small percentage of whom develop HTLV-1-associated myelopathy/tropical spastic paraparesis (HAM/TSP) or adult T-cell leukemia/lymphoma (ATLL). Patients with HAM/TSP suffer from progressively worsening myelopathic symptoms, such as motor disability and bladder dysfunction, and may become wheelchair-bound or even bedridden.

**Methods:**

To learn more about this rare, debilitating disease, we established the national registration system “HAM-net” in March 2012. We continuously obtain detailed data from enrolled patients using the registration forms and an annual telephone interview. In this retrospective study, we describe the demographics and clinical histories of 383 registered patients from all over Japan.

**Results:**

Patients were diagnosed at a median of 53 years old, long after disease onset at 45. Most (55.3 %) were originally from the southernmost regions, Kyushu and Okinawa. The main initial symptoms were difficulty walking (81.9 %), urinary dysfunction (38.5 %), and lower limb sensory disturbances (13.9 %). Many patients reported frequent leg numbness and leg pain, and the vast majority required medical intervention for urinary symptoms and constipation. A median of 8 years elapsed from the onset of motor symptoms to Osame Motor Disability Score (OMDS) 5 (requiring unilateral support), 12.5 years to OMDS 6 (requiring bilateral support), and 18 years to OMDS 9 (unable to walk). Health Assessment Questionnaire - Disability Index (HAQ-DI) tasks related to mobility, as opposed to hand motions, were very difficult for HAM/TSP patients and well-correlated with OMDS. Scores on the MOS 36-Item Short-Form Health Survey (SF-36) indicated that physical functioning was severely impaired in HAM/TSP patients. Patients with a history of blood transfusion (19.1 %) were older and suffered from more severe disability as indicated by their high HAQ-DI scores. Patients with a family history of HAM/TSP (8.4 %) were younger and had relatively mild symptoms given their long disease durations; many (15.6 %) also had a relative with ATLL.

**Conclusions:**

The HAM-net national registration system has been an effective tool for gathering personal and clinical data from HAM/TSP patients scattered throughout Japan. We expect to conduct many retrospective and prospective epidemiological studies using HAM-net in the future.

## Background

Human T-lymphotropic virus type 1 (HTLV-1) is a retrovirus mainly endemic to southwestern Japan, South America, sub-Saharan Africa, and the Caribbean islands. A recent report on global HTLV-1 epidemiology gave an estimate of only five to ten million HTLV-1-infected individuals worldwide [1]. However, the results were based only on endemic areas with reliable data, suggesting that the actual number may be much higher if countries such as China and India were also included. At least one million are estimated to reside in Japan [2], with prevalence rates differing widely among prefectures, ranging from less than 1 % in the north to more than 6 % in the south, where in certain towns as many as 30–40 % of adults aged 50 and over are infected [1]. While the vast majority of those infected remain lifelong asymptomatic carriers, 0.25–3 % develop HTLV-1-associated myelopathy/tropical spastic paraparesis (HAM/TSP) [3–5], 2–7 % develop adult T-cell leukemia/lymphoma (ATLL) [6–8], and some develop other inflammatory conditions such as uveitis, dermatitis, thyroiditis, pneumonitis, myositis, and arthritis [9].

Patients with HAM/TSP suffer from chronic spinal cord inflammation, experiencing progressively worsening myelopathic symptoms such as spastic paraparesis, lower limb sensory disturbances, and bladder and bowel dysfunction [10]. The disease severely impacts their quality of life, both physically and emotionally, mainly due to difficulties with locomotion, bladder management, and chronic pain [11, 12]. In the later stages of the disease, patients may become wheelchair-bound or even bedridden [13]. Therapies such as corticosteroids and interferons, which can tame the hyperactive immune response associated with HAM/TSP, reportedly produce modest benefits; however, long-term treatment is difficult to tolerate due to adverse effects that often rival the symptoms of the disease [14]. Moreover, due in part to the fact that patients with this rare disease are few and far between, there have been no proper placebo-controlled trials for interferons or for corticosteroids. Thus, the prognosis for HAM/TSP patients is very poor.

**Fig. 1:**
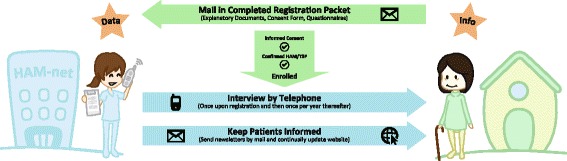
Enrollment process for the HAM-net registration system. This figure is displayed on the English version of the HAM-net website, hamtsp-net.com. The patient (right) must request a registration packet and mail the completed paperwork to the HAM-net offices, including documentation of the HAM/TSP diagnosis and a signed informed consent document. HAM-net staff (left) then contact the patient by telephone to conduct an interview every year. Data is obtained from both the registration documents and the telephone interviews. In return, patients are kept informed with regular newsletters and updates to the HAM-net website

In order to gain a clear understanding of the disease and its impact, it is necessary to learn more about HAM/TSP, including the frequency and severity of the symptoms, the speed of disease progression, and the attributes of the individuals suffering from the disease. Because HAM/TSP is such a rare disease, it is difficult to gather a cohort large enough for meaningful epidemiological analysis. Therefore, we have established a national registration system for HAM/TSP patients in Japan, known as “HAM-net” (Fig. 1). With this system in place, we can gather data from hundreds of HAM/TSP patients scattered all throughout Japan, including personal, demographic, and especially clinical data. There have been other studies describing the characteristics of patients with HAM/TSP in Japan [15–17] and elsewhere, such as in the UK [18], Martinique [19], Brazil [20, 21], Peru [22], and Iran [23], but no patient registries.

The unique design of HAM-net allows patients from all over the country to self-report their status in great detail at yearly intervals, allowing us to obtain volumes of both retrospective and prospective data. Since there is no standard protocol for monitoring HAM/TSP symptoms, we included several established scales and surveys in our patient interviews so that we may assess their applicability to HAM/TSP. HAM-net is flexible enough to evolve with new findings by adding or subtracting questions from the yearly interview if indicated. The HAM-net network is also a valuable resource for recruiting subjects for clinical studies. Moreover, enrolled patients benefit by regularly receiving information from our office, such as updates on our group’s activities and the progress of HAM/TSP research as well as useful resources. Our satisfaction surveys indicate that enrolled patients find our newsletters particularly helpful and desire frequent updates [24].

In this paper, we describe the establishment of the HAM-net patient registry and the first findings made using this system. These findings constitute the results of a retrospective epidemiological study in which data obtained from patients during the registration process and initial interview were evaluated to establish a detailed profile of patients with HAM/TSP in Japan. In addition, analysis was performed to investigate how factors such as history of blood transfusion and family history of HAM/TSP may correlate with disease severity; such results could yield insights into how the mode of HTLV-1 transmission or host genetics may play a role in disease progression.

## Methods

### Aims

The aims of this report are to introduce HAM-net, a novel HAM/TSP patient registration system, and announce the first findings produced using this system. HAM-net is intended to function primarily as a national database for patient information in order to determine: the profile of the Japanese HAM/TSP patient, the most effective methods for monitoring patient symptoms, and factors that affect disease progression. In the long-term, these findings will be used to help physicians more effectively diagnose HAM/TSP, monitor disease progression, and make patient-specific predictions on disease prognosis, ultimately improving the HAM/TSP treatment paradigm. Secondary purposes of HAM-net include establishing a contact database to recruit patients for clinical studies, raising awareness about HAM/TSP among physicians, and keeping patients well informed.

### Establishment

The HAM-net national registration system for HAM/TSP patients was established in March 2012 at the St. Marianna University School of Medicine in Kanagawa, Japan, where it is managed by the HAM/TSP research group in the Department of Rare Diseases Research. The management team includes scientists, physicians, nurses, professional clinical research coordinators, professional data managers and analysts, web administrators, and secretarial staff. The management team meets once per month to discuss progress and future directions. Collaborators include physicians from other institutions and HAM/TSP patient groups. This study is approved by the St. Marianna University School of Medicine Bioethics Committee (Approval ID No. 2044) and supported by the Practical Research Project for Rare/Intractable Diseases from the Japan Agency for Medical Research and Development (AMED).

### Patient recruitment

Patients diagnosed with HAM/TSP are recruited from all over Japan by ensuring that informational leaflets are distributed to patients at clinics, meetings of patient groups, and lectures. Using the Japanese Society of Neurology contact list, we sent information about HAM-net and a copy of the leaflet to all of the roughly 5000 board-certified neurologists in Japan. In addition, we asked the leader of the national HAM/TSP patient association to send the leaflets to all members of the group, which included roughly 300 patients. Information is also freely available online at the registry website, hamtsp-net.com.

### Patient registration

Patients wishing to register may apply to do so via telephone, FAX, or e-mail. The applicant then receives a packet of registration materials in the mail, including detailed explanations of the research objectives and risks of enrolling in the study, consent forms, and registration forms to be filled out by the applicant as well as by the attending physician, when possible (Fig. 1). The inclusion criteria for HAM-net are confirmed HAM/TSP diagnosis and informed consent, and there are no exclusion criteria. Once both informed consent and confirmation of diagnosis have been obtained, the applicant may be enrolled as a subject in the study. The subject may subsequently withdraw their consent and would be removed from the registry immediately upon request. Patients were informed that refusal to participate in the study would not affect their medical care. All subjects in this study willingly gave written informed consent.

### Data collection

Data is collected from registered patients using the registration forms followed by an in-depth telephone interview. The nurse or clinical research coordinator conducting the interview assesses the patient’s health and quality of life using various scales and surveys over the course of 45–60 min. The interviewers are instructed by the principal investigator and provided with a standard operating procedures manual such that the interviews are conducted in a uniform manner. This telephone interview is conducted once upon registration and once per year thereafter in order to observe each patient’s progress over time.

### Data storage

All patient information sent to our HAM-net Patient Registration Center is processed by the Personal Information Manager, who de-identifies the data by assigning each patient an anonymous ID number. The Personal Information Manager uses a single computer set aside and designated for this purpose only. Only this de-identified information is used for data analysis, and no identifying information will be published. The physical copies of the patients’ forms are stored under lock and key.

### Data contents

What follows is a brief overview of the types of data collected from patients:

1. Basic Information (Name, Birthdate, Gender, Residence).
2. Contact Information.
3. Personal and Financial Information (Occupation, Income, Employment Status, Marital Status, Family Structure, Welfare Status, Insurance Status, Disability Status).
4. Medical Records (Disease History, Treatment History, Complications, History of Blood Transfusions, Family History, Blood and Cerebrospinal Fluid Test Results – when obtainable from attending physician).
5. Scales and Surveys.
  - Osame Motor Disability Score (OMDS) – Score ranges from 0 to 13, where 0 indicates no motor disability and higher scores indicate increasingly severe motor disability [25]. See Table 2 for details.
  - Health Assessment Questionnaire – Disability Index (HAQ-DI)* – Scores range from 0 to 3 for each of 20 items (overall HAQ score is the average of these 20 scores), with higher scores indicating more severe disability in performing daily tasks [26].
  - Insituto de Pesquisa Clinica Evandro Chagas Disability Score (IPEC)-1 – Total score ranges from 0 to 29, with 17 possible points for motor score, 3 for spasticity score, 4 for sensory score, and 5 for sphincter score, with more points indicating more severe neurological disability [27, 28]. Here we report only on the gait disturbance subsection of the motor score, which ranges from 0 to 11, and on the contents of the sensory section, which describes lower limb pain and numbness.
  - International Consultation on Incontinence Questionnaire – Short Form (ICIQ-SF) – Scores range from 0 to 5, 6 (even numbers only), and 10 for urinary leak frequency, amount, and impact on daily life, respectively (total ICIQ-SF ranges 0-21), with higher scores indicating more severe incontinence [29]. There is also one unscored qualitative question on leakage circumstances.
  - International Prostate Symptom Score (I-PSS) – Scores range from 0 to 5 each for incomplete emptying, frequency, intermittency, urgency, weak stream, and straining (total I-PSS ranges 0–30), with higher scores indicating more severe symptoms [30].
  - Overactive Bladder Symptom Score (OABSS) – Scores range from 0 to 2, 3, 5, and 5 for daytime urinary frequency, nighttime frequency, urgency, and urgency incontinence, respectively (total OABSS ranges 0–15), with higher scores indicating more severe symptoms [31, 32].
  - Nocturia Quality of Life (N-QOL) – Scores range from 0 to 4 for each of 12 items concerning the impact of nocturia on quality of life; these scores are summed and transformed into a standardized scale from 0 to 100, with higher scores indicating better quality of life [33]. In addition, there is one global QOL item asked but not included in the N-QOL total score.
  - MOS 36-Item Short-Form Health Survey (SF-36) – The average score for Japanese people was set at 50 with 10 as the standard deviation, with higher scores indicating better health-related quality of life [34]. The eight categories assessed were: physical functioning, role-physical, bodily pain, general health, vitality, social functioning, role-emotional, and mental health.

### Statistical analysis

All analyses were descriptive and exploratory. Although *p* values were reported, no significance levels were set for inference, and all interpretations were descriptive. All reported *p* values were two-sided and unadjusted for multiplicity. Continuous and discrete variables were summarized using medians with interquartile ranges (IQR) or numbers of patients with percentages of total patients, respectively. Differences in continuous and discrete variables between two groups were assessed using the Mann-Whitney *U* test and the Chi-squared test, respectively. Correlations of two continuous variables were assessed using the non-parametric Spearman’s method. Calculations were made using IBM SPSS Statistics Version 22.0 software.

## Results

**Fig. 2:**
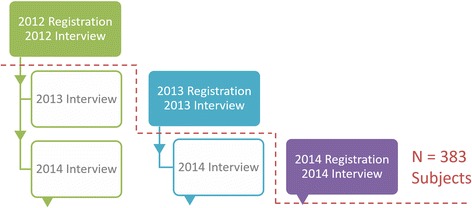
Registration and interview timeline for the HAM-net subjects analyzed in this study. Each colored column represents a group of patients who registered during the same year: 2012 (green), 2013 (blue), or 2014 (purple). Patients who registered in 2015 or later (not shown) were not included as subjects in this study. All subjects were interviewed once upon registration and then once per year thereafter; however, data from these follow-up interviews (shown in gray text) are not included in this paper. In total, data from three years of initial interviews for 383 subjects were analyzed in this retrospective study. A red dotted line separates the data that were and were not analyzed

Between March 2012 and March 2015, 515 individuals requested application materials for the HAM-net patient registration system, and 383 confirmed HAM/TSP patients proceeded through final registration and completed the initial interview (Fig. 2). Table 1 summarizes the results of these interviews.

**Table 1:** Clinical attributes of the HAM/TSP patients enrolled in this study (*n* = 383)

Time-Points (Years)		Median	(IQR)
Age at Onset		45	(32 – 56)
Age at Diagnosis		53	(42 – 62)
Age (at Present^a^)		63	(56 – 69)
Diagnosis Delay (Time from Onset to Diagnosis)		5	(1 – 12)
Disease Duration (Time from Onset to Present^a^)		17	(8 – 26)
Scales/Surveys (Scores)		Median	(IQR)
OMDS (Range: 0 – 13)		5.0	(5.0 – 7.0)
IPEC-1 Gait Subsection (Range: 0 – 11)		4.0	(3.0 – 8.0)
ICIQ-SF (Range: 0 – 21)		6.0	(0.0 – 11.0)
I-PSS (Range: 0 – 30)		14.5	(6.0 – 22.0)
OABSS (Range: 0 – 15)		6.0	(2.3 – 10.0)
N-QOL (Range: 0 – 100)		91.7	(74.5 – 100.0)
HAQ-DI (Range: 0 – 3)		1.1	(0.8 – 1.6)
SF-36 Physical Functioning (National Average ± SD: 50 ± 10)^b^		-3.5	(-10.7 – 14.5)
SF-36 General Health (National Average ± SD: 50 ± 10)^b^		38.9	(32.5 – 45.8)
Inquiries	Responses	Subjects	(Percentage)
Sex:	Female	284	(74.2 %)
	Male	99	(25.8 %)
Initial Symptoms (inclusive):	Gait Disturbance	313	(81.9 %)
	Urinary Disturbance	147	(38.5 %)
	Sensory Disturbance (in legs)	53	(13.9 %)
	Other	115	(30.0 %)
History of Blood Transfusion:	Yes (Total)	73	(19.1 %)
	Yes, Pre-1986	57	(14.9 %)
	Unknown	3	(0.8 %)
Urinary Dysfunction:	No Symptoms	29	(7.6 %)
	Symptoms or Using Medication	234	(61.3 %)
	Using Catheters	108	(28.3 %)
	Other Management Strategy	11	(2.9 %)
Bowel Dysfunction:	No Symptoms	87	(22.8 %)
	Symptoms or Using Medication	256	(67.0 %)
	Using Enemas	33	(8.6 %)
	Other Management Strategy	6	(1.6 %)
Leg Numbness:	None	125	(32.7 %)
	Occasional	73	(19.1 %)
	Severe	184	(48.2 %)
Leg Pain:	None	215	(56.3 %)
	Occasional	78	(20.4 %)
	Severe	89	(23.3 %)

Data are summarized using the median and interquartile range (IQR) or the number of subjects and the percentage of total subjects (%). The full names of the scales and surveys shown are as follows: Osame Motor Disability Score (OMDS), Health Assessment Questionnaire – Disability Index (HAQ-DI), Insituto de Pesquisa Clinica Evandro Chagas Disability Score (IPEC)-1, International Consultation on Incontinence Questionnaire – Short Form (ICIQ-SF), International Prostate Symptom Score (I-PSS), Overactive Bladder Symptom Score (OABSS), Nocturia Quality of Life (N-QOL), and MOS 36-Item Short-Form Health Survey (SF-36). Higher scores on N-QOL and SF-36 indicate better health, whereas the opposite is true for all other scales and surveys. n values vary between *n* = 363 and *n* = 383 due to incomplete data

^a^Present is defined as the time of the subject’s initial HAM-net interview

^b^The median values for the remaining six SF-36 sub-scores were all within one standard deviation of the national average (40 – 60) and are not shown in this table

### Demographics

**Fig. 3:**
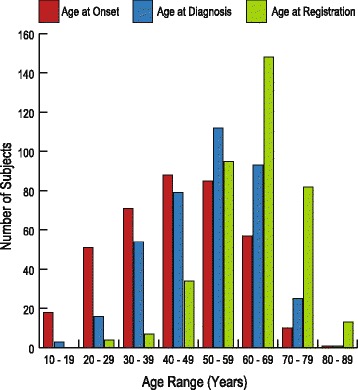
Age of the HAM-net subjects analyzed in this study. This graph illustrates the marked delay between age of onset (*n* = 381) and age at diagnosis (*n* = 383) for HAM/TSP patients and shows that HAM-net subjects have mostly been suffering from the disease for many years before registering. The most common age of onset was during the subjects’ 40s, but they were primarily diagnosed in their 50s. Nearly 40 % of HAM-net subjects are currently in their 60s, the largest HAM-net age demographic

Enrolled patients were a median (IQR) of 63 (56–69) years old at the time of registration and had been suffering from the disease for 17 (8–26) years (Fig. 3). The age of onset varied from 10 to 85 years old, with patients most commonly noticing their first symptoms in their 40s (23.1 %) and 50s (22.3 %). While the age of onset was 45 (32–56) years, the age at diagnosis was much later, at 53 (42–62) years of age. It took 5 (1–12) years from disease onset to diagnose HAM/TSP in Japan. Nearly three-quarters (74.2 %) of the patients were women, and there were no remarkable differences in age, age of onset, or age at diagnosis between men and women (data not shown).

**Fig. 4:**
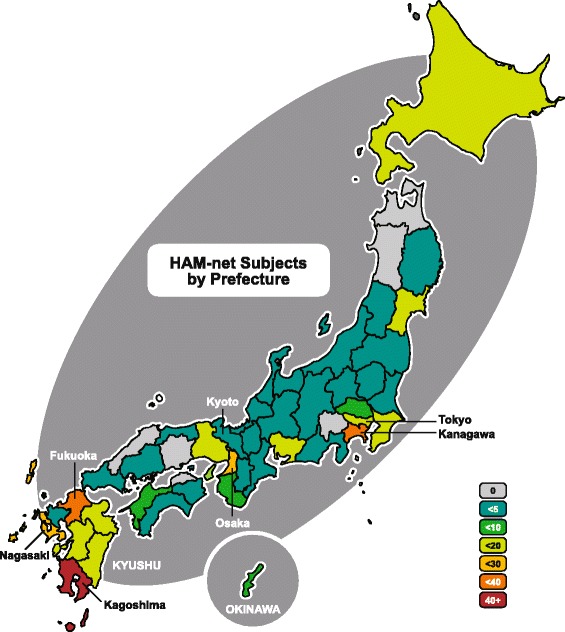
Population map for HAM-net subjects. Map shows the prefectures occupied by HAM/TSP patients enrolled in this study, color-coded according to the number of subjects (*n* = 383). Prefectures that are well-known reference points, such as Tokyo, or hot-spots for HAM-net enrollees, such as Kagoshima, are labeled. Subjects resided in all but six of the 47 prefectures in Japan

HTLV-1 is most prevalent in the southernmost islands, Kyushu and the Okinawa archipelago, and the eight prefectures composing those islands are considered endemic regions [1]. Most (212, 55.3 %) HAM/TSP patients enrolled in this study were originally from those regions, even if some (42) had since moved elsewhere. As shown in Fig. 4, enrolled patients resided in 41 of the 47 prefectures, illustrating how HTLV-1 has spread throughout Japan. The patient population spiked in the major metropolitan centers Tokyo (20, 5.2 %) and Osaka (24, 6.3 %), even though they are not located in the south. By contrast, Kyoto, a prefecture neighboring Osaka, was home to only 1 % of the enrolled patients. It should be noted that our research is based in Kanagawa prefecture, resulting in a disproportionately high number of patients (34, 8.9 %) being recruited in that area. Only 13 patients (3.4 %) were originally from Kanagawa.

### Symptoms

Patients with HAM/TSP are plagued with a variety of neurological symptoms, notably lower limb pain and sensory disturbances, urinary and bowel dysfunction, and motor disability. Impaired mobility can be measured using OMDS, a motor disability scale ranging from normal mobility at OMDS 0 up to complete paraplegia at OMDS 13 (Table 2) [25]. According to this study, the majority of HAM/TSP patients in Japan require some form of support to walk, most commonly a cane, but can still walk at least 10 m at a time with that support (OMDS 4–6, 63.0 % of patients). While 28.1 % of patients were more severely disabled (at or above OMDS 7), fewer than 5.0 % were bedridden (at or above OMDS 11). HAM-net subjects scored a median of 4.0 (3.0–8.0) out of 11.0 on the gait subsection of the IPEC-1 scale, which was designed to measure the presence of neurological symptoms in HAM/TSP patients [27]. Extracting sensory data from IPEC-1 revealed that leg numbness and pain heavily impact HAM/TSP patients: the majority of HAM-net subjects reported experiencing leg numbness, with 184 (48.2 %) indicating that the numbness occurred very often or always; roughly half of the patients had chronic leg pain, with 89 (23.3 %) reporting that they were very often or always in pain.

**Table 2:** Osame Motor Disability Score (OMDS) descriptions and number of HAM-net subjects (*n* = 382)

OMDS	Description	Subjects	(Percentage)
0	No walking or running abnormalities	3	(0.8 %)
1	Normal gait but runs slowly	5	(1.3 %)
2	Abnormal gait (stumbling, stiffness)	13	(3.4 %)
3	Unable to run	11	(2.9 %)
4	Needs handrail to climb stairs	47	(12.0 %)
5	Needs a cane (unilateral support) to walk	130	(34.0 %)
6	Needs bilateral support to walk	65	(17.0 %)
7	Can walk 5-10 m with bilateral support	29	(7.4 %)
8	Can walk 1-5 m with bilateral support	25	(6.5 %)
9	Cannot walk, but able to crawl	19	(5.0 %)
10	Cannot crawl, but able to move using arms	19	(5.0 %)
11	Cannot move around, but able to turn over in bed	3	(0.8 %)
12	Cannot turn over in bed	3	(0.8 %)
13	Cannot even move toes	10	(2.6 %)

Data are summarized using the number of subjects (percentage of total subjects). Scores listed are those at the time of the subject’s initial HAM-net interview

Urinary dysfunction and constipation were also widely reported among HAM/TSP patients, most of whom have taken steps to alleviate these troubling symptoms. At the time of this survey, as many as 234 (61.3 %) patients suffered from urinary symptoms and 256 (67.0 %) from constipation, many of whom were using medication to suppress their symptoms. In addition, several patients were forced to take more extreme measures: 108 (28.3 %) patients inserted catheters in order to urinate, and 33 (8.6 %) underwent enemas. There were no noticeable differences regarding how men versus women managed their symptoms. As shown in Table 1, scores on the scales and surveys specifically assessing urinary symptoms (ICIQ-SF, I-PSS, OABSS, and N-QOL) were varied.

HAM-net subjects reported that there were three main initial symptoms associated with the onset of HAM/TSP. Most but not all patients (81.9 %) noticed difficulty walking, many patients (38.5 %) experienced urinary dysfunction, and a substantial minority (13.9 %) suffered from lower limb sensory disturbances. Interestingly, this survey revealed that women were more likely than men to experience urinary dysfunction as an initial symptom (43.5 % vs. 24.2 %, respectively, *p* < 0.001). Moreover, 51 patients (13.4 %) complained of urinary issues before noticing any difficulty walking. The majority of these patients (43 of 51) were women, who were slightly more likely than men to exhibit this pattern (15.2 % vs. 8.1 %, respectively).

### Disease progression

**Fig. 5:**
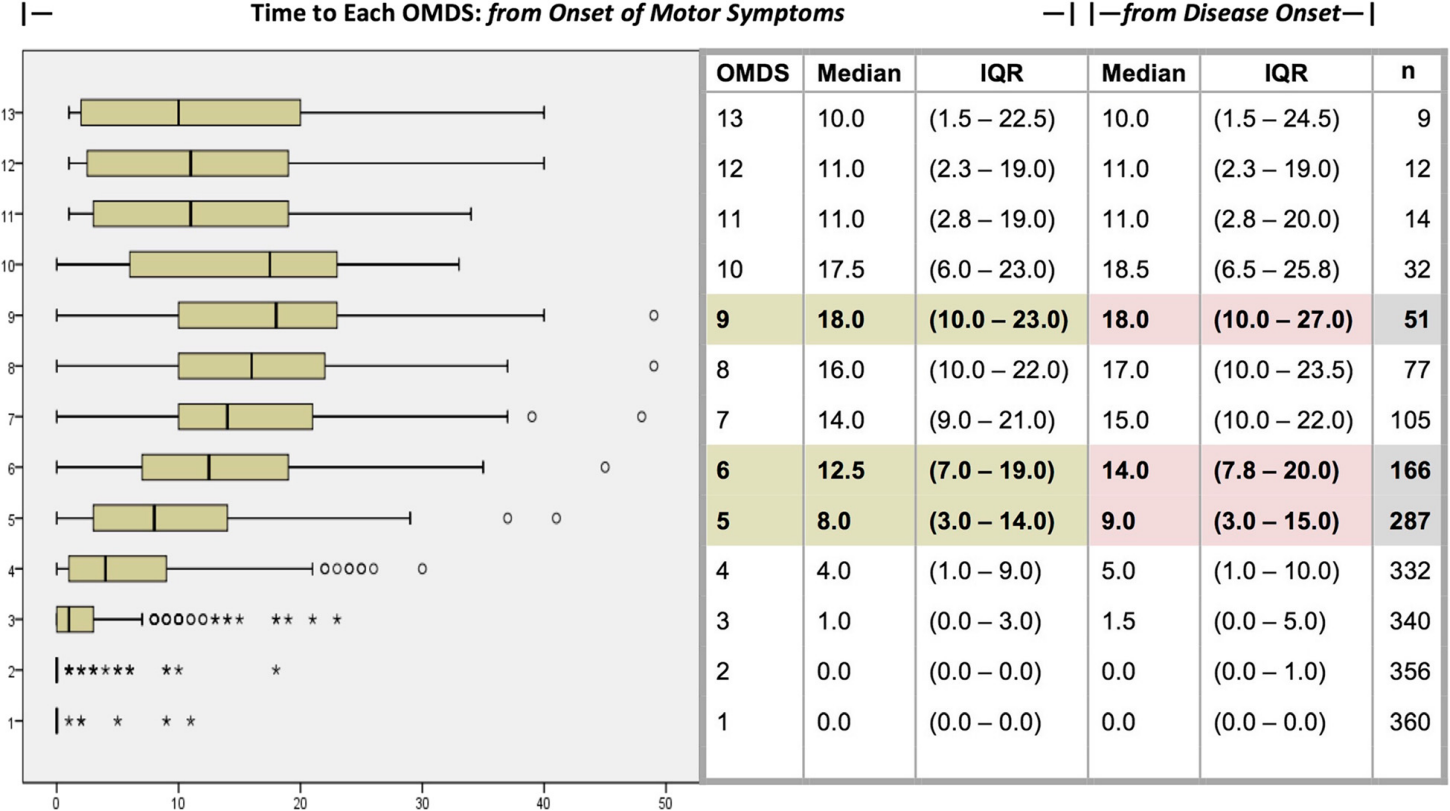
Rate of Motor Disability Progression. The table lists the time elapsed to progress to each Osame Motor Disability Score (OMDS) both from onset of motor symptoms and from disease onset for the HAM/TSP patients enrolled in this study based on retrospective data. The corresponding graph illustrates the times starting from onset of motor symptoms. The boxes encompass the 25^th^–75^th^ percentiles, the interquartile range (IQR), and the bars indicate the minimum and maximum values within 1.5 IQR of the box. Circles represent outliers beyond that range, and stars represent extreme outliers beyond 3 IQR. Importantly, the medians cease to increase linearly and begin to curve at around OMDS 9, suggesting variation in progression speeds. In the table, the values for OMDS 5, 6, and 9 are highlighted to emphasize those important time-points: need for unilateral support, bilateral support, and complete inability to walk, respectively

Changes in OMDS over time, assessed retrospectively by conducting a detailed patient history, were used to estimate the rate of disease progression (Fig. 5). A median of 8 (3–14) years elapsed from the onset of motor symptoms to OMDS 5 (requiring unilateral support to walk, e.g. a cane), 12.5 (7–19) years to OMDS 6 (requiring bilateral support to walk, e.g. a walker), and 18 (10–23) years to OMDS 9 (unable to walk at all). As illustrated in Fig. 5, the median scores increased steadily until OMDS 9, at which point they began to decrease again. We also recorded changes in OMDS over time starting from disease onset: 9 (3–15) years elapsed from disease onset to OMDS 5, 14 (7.5–20) years to OMDS 6, and 18 (10–27) years to OMDS 9.

### Disease impact

**Fig. 6:**
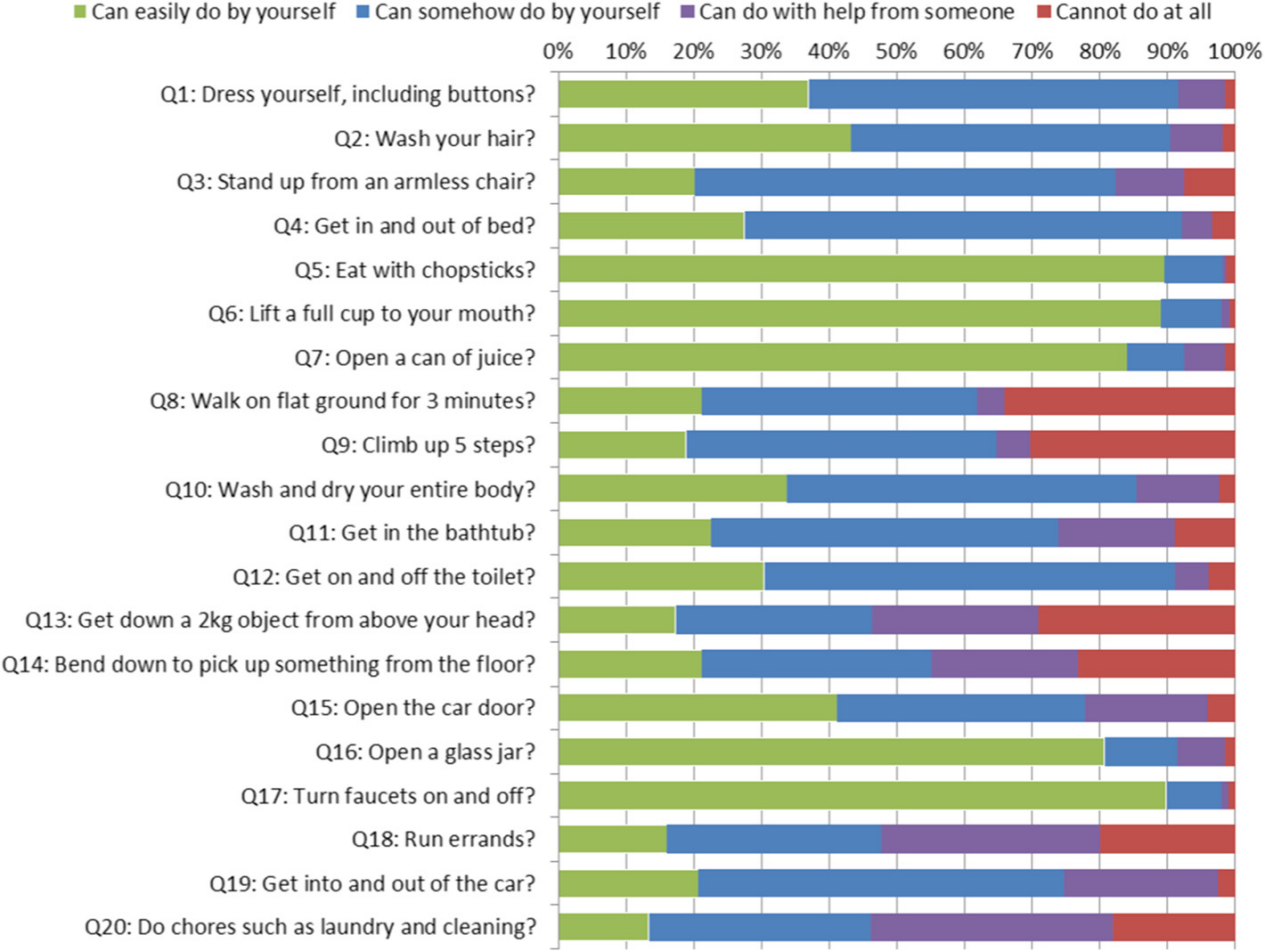
Results of the Health Assessment Questionnaire – Disability Index (HAQ-DI). HAM/TSP patients enrolled in HAM-net reported the level of difficulty they experienced performing each of the 20 HAQ-DI tasks, listed in the figure as Q1-Q20 (*n* = 382)

**Fig. 7:**
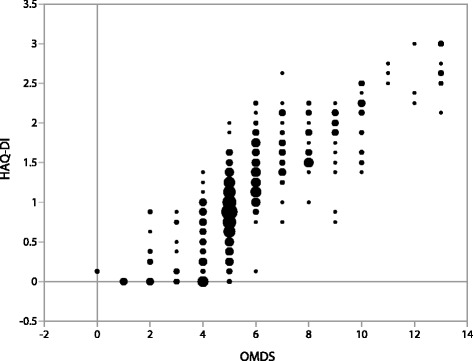
Correlation between motor disability and activities of daily life. Figure shows the correlation between the Osame Motor Disability Score (OMDS) and the average Health Assessment Questionnaire – Disability Index (HAQ-DI) scores for the HAM/TSP patients enrolled in this study (*n* = 382). Circles are sized according to the number of patients at each data point, with the smallest circles indicating a single patient each and the largest circles indicating 22 patients. The correlation coefficient was ρ = .832

Disability and pain impact the so-called Activities of Daily Living (ADL), and we assessed this impact using HAQ-DI. HAM/TSP patients scored a median of 1.1 (0.8–1.6) total (Table 1). Detailed analysis revealed that they exhibited the most difficulty accomplishing tasks specifically requiring mobility, such as walking around (Q8) and climbing stairs (Q9), which were completely impossible for 34.0 % and 30.4 % of patients, respectively (Fig. 6). Also frequently impossible were tasks related to reaching up (Q13, 29.1 %) or down (Q14, 23.3 %) to retrieve objects as well as very active endeavors such as running errands (Q18, 19.9 %) and doing chores (Q20, 18.1 %). The vast majority of patients could complete tasks related to personal grooming (Q1-2) and hygiene (Q10-12) as well as getting in and out of bed, chairs, and the car (Q3, 4, 19), but most experienced some difficulty performing these tasks independently. On the other hand, tasks involving mainly hand motions such as eating (Q5-7) and gripping (Q16-17) could be performed easily by over 80 % of patients. Total HAQ-DI score was very well-correlated with OMDS (*ρ* = .832, *p* < 0.001, Fig. 7). Tasks involving heavy use of the lower limbs (Q3, 8, 9, 13, 19) were well-correlated with OMDS (*ρ* = 0.7), whereas the five tasks involving only hand motions (Q5, 6, 7, 16, 17) showed very little correlation (*ρ* < 0.3).

In addition, the SF-36 survey was used to assess the health-related quality of life of HAM/TSP patients relative to that of average Japanese citizens, whose scores were set to 50 with a standard deviation of 10 (Table 1). The average (and median) scores for six of the eight sub-sections (role-physical, bodily pain, vitality, social functioning, role-emotional, and mental health) were within one standard deviation of normal (between 40 and 60, data not shown). The score for general health was just barely lower, at a median of 38.9 (32.5–45.8). The only strikingly impaired section was physical functioning, where HAM/TSP patients scored a median of -3.5 with a wide IQR (−10.7–14.5).

### Blood transfusion history

Of the patients enrolled in this study, 73 (19.1 %) had a history of receiving blood transfusions, 57 of whom received blood before HTLV-1 screening for blood donations was implemented in 1986. Of the 16 patients who received blood after 1986, 10 were from Kyushu or Okinawa, areas where HTLV-1 is endemic. Those who had received blood tended to be older than those who had not: median ages at onset, diagnosis, and present were five to six years later (Table 3). In addition, although they had similar, even slightly shorter, disease durations, they experienced more severe disabilities as indicated by higher HAQ-DI scores: 1.3 (0.9–1.9) versus 1.0 (0.6–1.6).

**Table 3:** Variation in clinical attributes with history of blood transfusion (*n* = 380)

Attribute		Transfusion (+) (*n* = 73, 19.2 %)		Transfusion (-) (*n* = 307, 80.8 %)		*p* Value
Sex:	Female	58	(79.5 %)	224	(73.0 %)	0.255
	Male	15	(20.5 %)	83	(27.0 %)	
Age at Onset		50	(39 – 61)	44	(32 – 55)	0.005
Age at Diagnosis		57	(48 – 67)	52	(41 – 61)	0.004
Age (at Present^a^)		69	(58 – 74)	63	(56 – 68)	0.001
Diagnosis Delay (Time from Onset to Diagnosis)^b^		5	(1 – 12)	5	(1 – 12)	0.730
Disease Duration (Time from Onset to Present^a^)^c^		16	(8 – 23)	17	(9 – 26)	0.403
OMDS (Range: 0 – 13)		5.0	(5.0 – 7.5)	5.0	(5.0 – 7.0)	0.212
HAQ-DI (Range: 0 – 3)		1.3	(0.9 – 1.9)	1.0	(0.6 – 1.6)	0.022

Data are summarized using the number of subjects (percentage of total subjects) or the median (interquartile range). The full names of the scales and surveys shown are Osame Motor Disability Score (OMDS) and Health Assessment Questionnaire – Disability Index (HAQ-DI). Higher scores indicate more severe disability. The values for time-points are given in years, and scale and survey results are reported as scores

^a^Present is defined as the time of the subject’s initial HAM-net interview

^b^Transfusion (+) *n* = 72, (-) *n* = 306

^c^Transfusion (+) *n* = 72

### Family history of HTLV-1-associated diseases

Several of the enrolled patients had family members who were also suffering from HAM/TSP. Although HAM/TSP is estimated to occur in as few as 0.3 % of individuals infected with HTLV-1 [3], as many as 8.4 % of enrolled patients reported having first- or second-degree relatives who also had HAM/TSP. First-degree relatives are parents, siblings, and children; second-degree relatives are aunts, uncles, nieces, nephews, grandparents, grandchildren, and half-siblings. In addition, 5.7 % of HAM/TSP patients had a family history of ATLL.

The features of these 32 familial cases were compared with those of the remaining 351 patients (Table 4). The patients with familial HAM/TSP were markedly younger, with a median age of onset 13.5 years earlier and age at diagnosis 15 years earlier. However, there was no evidence that they were diagnosed any more quickly; in fact, the median delay between onset and diagnosis was slightly longer for familial cases: 6 (2–12) versus 5 (1–12) years. Patients with familial HAM/TSP had been suffering from the disease for more extended periods of time than their non-familial counterparts: 23.5 (14–32) vs. 16 (8–25) years. Despite these longer disease durations, they did not exhibit more severe symptoms: there were no remarkable differences in OMDS, HAQ-DI scores, urinary and bowel symptom severity, or frequency of lower limb pain and numbness. Those with a family history of HAM/TSP were also more likely to have a family history of ATLL: 15.6 % of patients from the familial HAM/TSP group had a first- or second-degree relative with ATLL, whereas only 4.8 % of the non-familial HAM/TSP group had such a relative.

**Table 4:** Variation in clinical attributes with family history of HAM/TSP (*n* = 383)

Attribute		Familial HAM/TSP (*n* = 32, 8.4 %)		Non-Familial HAM/TSP (*n* = 351, 91.6 %)		*p* Value
Sex:	Female	19	(59.4 %)	265	(75.5 %)	.046
	Male	13	(40.6 %)	86	(24.5 %)	
History of ATLL^a^:	Yes	5	(15.6 %)	17	(4.8 %)	.012
History of Blood Transfusion:	Yes (anytime)	2	(6.3 %)	71	(20.4 %)	.052
	Yes, before 1986	2	(6.3 %)	55	(15.8 %)	.147
Age at Onset		32.5	(25 – 45)	46	(34 – 56)	.000
Age at Diagnosis		40	(30 – 54)	55	(44 – 62)	.000
Age (at Present^b^)		58	(52 – 66)	64	(56 – 70)	.018
Diagnosis Delay (Time from Onset to Diagnosis)		6	(2 – 12)	5	(1 – 12)	.765
Disease Duration (Time from Onset to Present^b^)		23.5	(14 – 32)	16	(8 – 25)	.002
OMDS (Range: 0 – 13)		5.0	(5.0 – 8.0)	5.0	(5.0 – 7.0)	.481
HAQ-DI (Range: 0 – 3)		0.9	(0.5 – 1.9)	1.1	(0.8 – 1.6)	.677

Data are summarized using the number of subjects (percentage of total subjects) or the median (interquartile range). The full names of the scales and surveys shown are Osame Motor Disability Score (OMDS) and Health Assessment Questionnaire – Disability Index (HAQ-DI). The values for time-points are given in years, and scale and survey results are reported as scores. *n* values vary between *n* = 379 and *n* = 383 due to incomplete data

^a^History of ATLL indicates the subject has a first- or second-degree relative with adult T-cell leukemia/lymphoma (ATLL)

^b^Present is defined as the time of the subject’s initial HAM-net interview

## Discussion

To the author’s knowledge, HAM-net is the first HAM/TSP patient registry in the world. As an epidemiological study of HAM/TSP patients, we believe ours is the first of its size to obtain such a detailed history by communicating directly with each subject. Moreover, a nation-wide study has not been conducted in Japan since 1990, when Osame et al. mailed surveys to medical institutions around the country [16], and after 25 years it was necessary to reevaluate the profile of the Japanese HAM/TSP patient.

### Diagnosing HAM/TSP and monitoring symptoms

Our finding that patients most commonly experience HAM/TSP onset in their 40s (Fig. 3) is consistent with the findings of previous studies conducted in Japan [15, 16]. However, we revealed for the first time that there is a long delay between disease onset and diagnosis in Japan: on average, there was a gap of 7.6 years (median 5, range 0–49, 95 % CI 6.8–8.4 years). For comparison, the gap was reported to be 5.3 years (median 3, range 0–33, 95 % CI 2.2–5.5 years) in Martinique [19] and 3.8 years (median 2, range 1–19 years) in the UK [18]. This discrepancy may be partially due to the fact that this national study includes patients diagnosed in rural areas with limited access to professionals familiar with this disease (Fig. 4). All over the world, many patients must endure a long struggle with mysterious symptoms before they are finally given a correct diagnosis, causing psychological strain and perhaps lowering their chances of managing the disease effectively [35]. Experts recommend that all spastic paraparesis patients in or near endemic areas be screened for HTLV-1 to ameliorate this problem [36, 37].

There is no standard system for monitoring the symptoms of HAM/TSP patients, and it is difficult to compare results across studies, especially internationally. One of the ultimate goals of HAM-net is to establish an effective system for physicians to track disease progress and for researchers to compare their findings. To work towards this goal, we have been gathering data using several pre-established scales and surveys as well as questions of our own design.

OMDS has been used for decades to monitor HAM/TSP patients [25], and in this study it was our primary means of measuring motor disability (Table 2, Fig. 5). We also collected similar data using the gait disturbance subsection of the IPEC-1 scale, named after the Evandro Chagas Institute of Clinical Research/Instituto de Pesquisa Clinica Evandro Chagas (IPEC) in Brazil, which was recently drafted to tally the presence and severity of HAM/TSP neurological symptoms including motor, spasticity, pain, numbness, urinary, and bowel symptoms [27]. It has been employed in a small clinical study in the UK [28]. We intend to evaluate the usefulness of IPEC-1 relative to OMDS in a future study. In this study, we reported the IPEC-1 gait sub-score and extracted data on lower limb numbness and pain shown in Table 1, finding that roughly half of all patients experienced some degree of lower limb pain and most experienced numbness.

Our questionnaire revealed that the majority of Japanese HAM/TSP patients require medical intervention to alleviate their urinary and bowel symptoms. Scores on the ICIQ-SF, I-PSS, OABSS, and N-QOL scales and surveys also indicated impaired urinary function; however, the scores were varied, reflecting differences in the nature of each questionnaire. For example, ICIQ-SF and N-QOL assess impact on quality of life, whereas I-PSS and OABSS focus on the severity of symptoms. Patients using unpleasantly aggressive means to treat their symptoms, such as catheters, may have eased their symptoms while decreasing quality of life. To the authors’ knowledge, this is the first time scores on ICIQ-SF, I-PSS, or OABSS have been reported for HAM/TSP patients. N-QOL was used as an outcome measure in a Japanese clinical trial for HAM/TSP in 2013 [38]. In future studies, we plan to use HAM-net data to compare the usefulness of these methods for monitoring urinary symptoms in HAM/TSP patients. Our findings demonstrate that although urinary and bowel symptoms are less visible, they are highly prevalent and severe in HAM/TSP patients, and it is critical that they not be overlooked by physicians during diagnosis, monitoring, and treatment.

Similar findings in South America have prompted researchers there to stress the importance of urinary complaints for the early diagnosis of HAM/TSP [20, 22]. Though they reported that the diagnosis of HAM/TSP tends to be delayed even longer for patients complaining first of urinary symptoms [22], we did not observe any difference in time from onset to diagnosis in this study (data not shown). We did, however, note that a fraction of patients experienced urinary symptoms before the onset of motor symptoms and that women were more likely than men to experience urinary dysfunction as an initial symptom. Given this data, doctors in endemic areas should be made aware that HAM/TSP may initially present as a problem with urinary function, especially in women.

HAM/TSP is well known to be two to three times more common in women than men [10], and as expected, nearly three-quarters of our subjects were female. This is thought to be because sexual transmission of HTLV-1 mainly occurs from male to female [1]; in addition, there is some evidence that the lifetime risk of developing HAM/TSP in HTLV-1-positive individuals is higher in women than men, although it is unclear why this would be the case [4].

### Estimating the rate of disease progression

Measuring changes in OMDS over time is an established method of estimating the rate of HAM/TSP progression. In this study, we recorded the number of years that elapsed between the onset of motor symptoms and progression to each OMDS reached so far by every patient who could recall this information (*n* = 360, Fig. 5). The results are consistent with the prevailing theory that while HAM/TSP is generally a slowly progressive disease, there is significant variation in progression speed, including a subset of patients who progress very rapidly. Figure 5 shows that the IQRs are quite large, illustrating this wide variation. Moreover, the medians increase steadily at first but then begin to curve around OMDS 9, presumably because only the group of patients who progress at the fastest rate ever reach the highest OMDS grades. We intend to evaluate progression speed in great detail in a future study.

Other studies have used similar techniques to evaluate the speed of motor disability progression in HAM/TSP patients; however, many of these studies use disease onset rather than onset of motor symptoms as a starting point. Using disease onset yields slightly longer time estimates because some patients experience urinary symptoms before motor symptoms begin. Therefore, for the sake of comparison, we also recorded changes in OMDS over time starting from disease onset: a median of 9 (3–15) years elapsed from disease onset to OMDS 5 (requiring unilateral support to walk), 14 (7.5–20) years to OMDS 6 (requiring bilateral support to walk), and 18 (10 – 27) years to OMDS 9 (unable to walk at all). Interestingly, Nakagawa et. al. reported in 1995 that it took only 12.4 ± 10.7 years (mean ± standard deviation) to progress from onset of disease to inability to walk [15]. Their study was large but only evaluated patients in Kagoshima prefecture, whereas the present study includes patients from 41 prefectures. Although there are no other large-scale studies from Japan on this topic, reports from overseas are also available for comparison. In the UK, Martin et al. observed that the median times from disease onset to dependency on a unilateral walking aid and subsequently a wheelchair were 11 years (95 % confidence interval: 2.8 to 17.30) and 18 years (95 % CI: 14 to 22), respectively [18]. In Martinique, Olindo et al. reported only 6 years (95 % CI: 5 to 7) from onset to unilateral aid, 13 years (95 % CI: 10 to 17) to bilateral aid, and as long as 21 years (95 % CI: 14 to 28) to wheelchair-dependency [19]. It should be noted that many of our wheelchair-bound patients are classified as well below OMDS 9 because they are still able to stand up from their wheelchairs and walk short distances with support. Thus, these values may not be directly comparable. It would benefit HAM/TSP research if there were an internationally agreed-upon standard for evaluating motor disability in HAM/TSP patients.

### Assessing disease impact

We assessed the impact of HAM/TSP on ADL using HAQ-DI (Fig. 6), a scale typically used for chronic rheumatic conditions such as arthritis [26]. To the authors’ knowledge, this is the first time the HAQ-DI scores of HAM/TSP patients have been reported. Since total HAQ-DI score was very well-correlated with OMDS, we inferred that this score could indeed be a useful measure for disease severity in HAM/TSP patients. Moreover, there were many cases where a difference in severity was detected using HAQ-DI but not OMDS, and vice versa, suggesting that combining these two measures could allow for more precise tracking of disease progression (Fig. 7). Importantly, OMDS was better correlated with certain HAQ-DI tasks than with others. Since tasks requiring heavy use of the lower limbs were particularly strenuous for HAM/TSP patients, it is of little surprise that those tasks were well-correlated. By contrast, the five tasks that focused on hand movements showed very little correlation with OMDS. Thus, it may be beneficial to exclude these five tasks (Q5, 6, 7, 16, 17) if in the future we create a HAM/TSP-specific ADL scale incorporating HAQ-DI elements.

We also assessed the impact of HAM/TSP on health-related quality of life using the SF-36 survey. Scores on this survey indicated that HAM/TSP severely impairs physical functioning but does not have a striking effect on other aspects of quality of life, such as social, emotional, and mental health. This may be due to the chronic, slowly progressive nature of the disease, which allows patients time to gradually adjust to their circumstances. However, there was a wide range of individual variation, and it is likely that patients in the advanced stages of HAM/TSP suffer from more quality of life deficits than the average patient. We intend to perform further analysis in future studies, determining what factors affect quality of life most and which SF-36 sub-sections are most useful for monitoring HAM/TSP patients.

### Importance of blood transfusion history

HTLV-1 can be transmitted by blood transfusion, but this risk has been considered relatively negligible since 1986, when nationwide screening of blood donors for HTLV-1 was implemented in Japan [16, 39]. As illustrated by the distribution of HAM-net subjects shown in Fig. 4, HTLV-1-associated disease has spread all throughout Japan; however, our data indicates that blood donor screenings have been effectively limiting the spread via contaminated blood since 1986. In 1990, Osame et al. found that 23.8 % of HAM/TSP patients had a history of blood transfusion but that the number of patients developing transfusion-associated HAM/TSP had already dropped precipitously since 1986 [16]. For comparison, they conducted surveys among random Kagoshima prefecture residents and hospitalized neurological patients, and they found that the frequencies of blood transfusion history in these control populations were 3 and 5 %, respectively, when age- and sex-matched to the HAM/TSP patient group. If the 57 patients who received blood before 1986 are excluded from the analysis, only 5.0 % (16/323) of our patients had a history of blood transfusion. Thus, although these data are not directly comparable, it is clearly possible that some or all of these transfusions were merely coincidental. Moreover, 10 of those 16 patients who received blood post-1986 originated from HTLV-1-endemic areas in southern Japan (Kyushu and Okinawa), where the risk of sexual and vertical transmission is relatively high. Therefore, we have inferred that the 1986 measures have likely been effectively limiting the spread of HTLV-1 via blood transfusion.

Patients with a history of blood transfusion (*n* = 73), as compared to those without (*n* = 307), exhibited more severe symptoms (Table 3). This may be due to an elevated immune response following blood transfusion [40]. To the authors’ knowledge, this is the first time such a trend has been demonstrated. In fact, previous studies have reported that history of blood transfusion does not impact disease severity or progression speed [15, 17]. Those studies did however agree with our result that transfusion recipients were older than their non-recipient counterparts, which may also have contributed to their relatively poor outcomes [15, 22]. In fact, since HTLV-1 screening for blood transfusions was implemented in 1986, it could be argued that most patients who were infected by contaminated blood must be old enough to have received a transfusion at least 30 years ago. These patients might then suffer from more severe symptoms merely due to their age, not due to the mode of transmission. It may be possible to do an age-matched comparison in the future.

### Importance of family history

Our finding that 8.4 % of our subjects had a first- or second-degree relative with HAM/TSP is consistent with the findings of Osame et al., who reported that 8 % of HAM/TSP patients had a sibling, parent, or grandparent with HAM/TSP [16]. This is considered a relatively high rate of familial outbreak given that only 0.3 % of HTLV-1 carriers are estimated to develop HAM/TSP in their lifetimes [3]. We also evaluated the attributes of patients with a family history (Table 4), and our results agreed with those of previous studies, which showed that familial cases of HAM/TSP present with a younger age of onset and a slower rate of progression compared to sporadic cases [25, 41]. Importantly, this study revealed that those with a family history of HAM/TSP are also more likely to have a family history of ATLL. To the authors’ knowledge, this is the first time such a trend has been reported.

The relatively high rates of disease outbreak within families may indicate that genetic risk factors play a role in determining whether an HTLV-1 infection will develop into a serious illness. Indeed, genetic traits, namely certain HLA alleles, have been reported to confer increased or decreased susceptibility to HAM/TSP [42, 43] or ATLL [44], albeit confined to specific ethnic groups [45, 46]. We know of no genetic markers reported to affect susceptibility to both diseases.

An alternative line of reasoning involves considering that family history of HTLV-1-associated diseases implies vertical transmission of HTLV-1, i.e. mother-to-child, mostly through breastfeeding [47]. Supporting evidence for this reasoning includes that early infection would explain the comparatively younger ages of onset in the patients with familial HAM/TSP. It has been established that ATLL is linked to HTLV-1 infection in early childhood via vertical transmission, which is said to allow time for ATLL to develop after its typical latency period of multiple decades [48, 49]. Unlike ATLL, HAM/TSP is more commonly associated with HTLV-1 infection acquired during adulthood, either via sexual transmission or blood transfusion [49]; however, vertical transmission of HTLV-1 can also lead to HAM/TSP [50, 51]. Bartholomew et al. reported that 97 % of the mothers of their ATLL patients and 33 % of the mothers of their HAM/TSP patients were seropositive for HTLV-1 [51]. Thus, given the strong association between vertical transmission and ATLL, and assuming that cases of familial HAM/TSP are largely cases of vertical transmission, it logically follows that a high percentage of these patients would have a family history of ATLL in agreement with our results.

Similarly, the relatively high percentage of patients with a family history of HAM/TSP (8.4 %) compared to the rate of HAM/TSP in the general HTLV-1-infected population (0.3 %) could be explained if vertical transmission of HTLV-1 conferred greater susceptibility to HAM/TSP than sexual transmission, which is the most common transmission route [49]. Much about HAM/TSP pathogenesis is still unknown, and it is possible that the opportunity for the infection to develop over several decades could increase the likelihood of developing HAM/TSP as well as ATLL. The latency period for HAM/TSP can also be a long as multiple decades [50], which suggests that early infection would increase the likelihood of developing the disease within the carrier’s lifetime. This theory is only speculation, and there is no evidence to directly support the claim that vertical transmission increases susceptibility to HAM/TSP. However, indirect evidence abounds. Studies have shown that HTLV-1 carriers with a family history of HAM/TSP or ATLL have significantly higher proviral loads [52, 53]. Moreover, animal experiments have directly shown that vertical transmission (specifically oral transmission) of HTLV-1 results in elevated proviral load [54, 55]. Finally, it is well established that high proviral load is associated with the development of HAM/TSP [56]. Thus, it is reasonable to speculate that vertical transmission of HTLV-1 may result in young HTLV-1 carriers with comparatively high proviral loads who are more likely to develop HAM/TSP than those infected sexually during adulthood. Those who develop HAM/TSP would belong in our familial HAM/TSP patient group and likely present as younger and with milder inflammatory symptoms either due to their youth or greater tolerance for the virus afforded by vertical transmission [57]; this prediction is consistent with our results. Thus, the authors propose that a future study should be carried out to assess whether vertical transmission in infancy confers a greater susceptibility to HAM/TSP than sexual transmission in adulthood, as is the case for ATLL.

### Limitations of this study

As a retrospective study based largely on patient recollections, our findings were vulnerable to recall error. Subjects may have mistakenly reported inaccurate information. Many subjects were elderly and did not have access to past medical records for reference. In future HAM-net studies, we will be able to rely more heavily on prospective analysis using data from the yearly follow-up interviews.

As with any registry, there is also the problem of recruitment bias. In order to recruit a sample representative of the general population in Japan, we sent information to all board-certified neurologists in the country, and patients were recruited from 41 of the 47 prefectures (Fig. 4). Still, a disproportionate number of patients were recruited from Kanagawa prefecture, presumably because our own HAM/TSP outpatient clinic recruits more effectively. It is also possible that many who live in rural areas did not seek medical advice for their HAM/TSP symptoms, were not diagnosed, and thus did not have the chance to learn about HAM-net, reducing our registry’s coverage. Another issue is that patients with a family history may have been more likely to consult a neurologist at the first sign of symptoms and thus more likely to join HAM-net. However, our data indicates that those with familial HAM/TSP were not diagnosed any more quickly than those without a family history (Table 4).

It is impossible to calculate the percentage of Japanese HAM/TSP patients who are enrolled in HAM-net, i.e. the coverage of the registry, because there is no official estimate of the number of HAM/TSP patients in Japan. In 1990, Osame et al. conducted a national survey and reported that there were at least 589 cases of HAM/TSP in Japan, while acknowledging that this was almost certainly a dramatic underestimate [16]. If it is true that there are 1.08 million people infected with HTLV-1 in Japan [2] with a 0.25 % lifetime incidence of HAM/TSP [3], there would necessarily be fewer than 2700 Japanese HAM/TSP patients. Considering these data as well as unofficial reports from colleagues, the authors speculate that there may be roughly 2000–2500 individuals suffering from HAM/TSP in Japan. With 383 subjects, this study would have thus enrolled about 15–20 % of the target population.

### Future directions

As mentioned, we plan to address certain topics in greater detail in future studies. To learn more about the variation in the rate of HAM/TSP progression, we intend to divide patients into groups based on progression speed and compare attributes between groups. We also plan to analyze which scales and surveys best reflect differences in the symptoms of HAM/TSP patients and eventually establish an effective monitoring method. In addition, we will be able to conduct prospective studies using data from yearly follow-up interviews; for example, we will be able to monitor the effects of treatments such as oral steroids over time. Finally, not only are we continuing yearly follow-up interviews for the current HAM-net subjects, but we are still accepting applications for new enrollees, and we expect the reach of HAM-net to expand over time. Since the data for this study was obtained and analyzed last year, we have already enrolled more patients, bringing our total up to 467 registered HAM-net subjects as of March 2016.

## Conclusions

By establishing a registration system for HAM/TSP patients, we have created an invaluable resource for epidemiological analysis. We were able to determine the demographics of HAM/TSP patients in Japan, including gender, age, and geographic location, giving us a better understanding of our patient population. Analysis of symptoms, such as motor, urinary, bowel, and sensory dysfunction, as well as disease impact, gave us new insights into the patient experience. Our examination of the course of the disease and the factors that influence it is expected to provide physicians with the means to make more accurate prognoses based on age, history of blood transfusion, and family history of HTLV-1-associated diseases. Finally, as is often the case with epidemiological studies, we found patterns that should inspire researchers to uncover new information about the pathogenesis and treatment of this disease, namely investigating if and how the mode of HTLV-1 transmission affects patient prognosis.

## Abbreviations

ADL: Activities of Daily Living
ATLL: adult T-cell leukemia/lymphoma
HAM/TSP: HTLV-1-associated myelopathy/tropical spastic paraparesis
HAQ-DI: Health Assessment Questionnaire – Disability Index
HTLV-1: human T-lymphotropic virus type 1
ICIQ-SF: International Consultation on Incontinence Questionnaire – Short Form
IPEC-1: Insituto de Pesquisa Clinica Evandro Chagas Disability Score 1
I-PSS: International Prostate Symptom Score
IQR: Interquartile range
N-QOL: Nocturia Quality of Life
OABSS: Overactive Bladder Symptom Score
OMDS: Osame Motor Disability Score
SF-36: MOS 36-Item Short-Form Health Survey
